# Reply to the letter concerning Kawasaki disease and Covid-19

**DOI:** 10.1017/S1047951120002231

**Published:** 2020-07-09

**Authors:** Rohit S. Loomba, Enrique Villarreal, Saul Flores

We thank Dr Wiwanitkit for his comment on our manuscript.^1^ He raises some valid points.

He notes that there was no report on Kawasaki-like syndrome in Covid-19 patients during the first phase of the pandemic in East Asia and Southeast Asia. Indeed, this is quite interesting. While we cannot comment with any certainty as to why this is the case there are a few things to keep in mind: (1) Kawasaki disease (KD) does have widely variable presentation in different ethnic groups and it could be that different triggers are more potent for different ethnic groups; (2) reports from Europe and the United States also had a lag from the initial rise in cases which may be attributed to the lower infection rate in children further compounded by the low incidence of KD with viruses in general; (3) lack of reporting does not necessarily imply that it did not occur sooner but that it simply was not reported sooner.^2^ Indeed, Dr Wiwanitkit posits the first of these possibilities in his commentary, further, by stating the documented phenomenon of differences in immunoglobulin responsiveness amongst various ethnic groups.^3,4^

Differences in KD presentation have been demonstrated by age, ethnic group, and by those who present with macrophage activation syndrome versus those who do not.^2,5^ This hyperinflammatory response noted in children with exposure to Covid-19 likely represents KD with macrophage activation syndrome. Macrophage activation syndrome is often triggered by infectious or rheumatologic conditions, most commonly juvenile idiopathic arthritis. Nonetheless, it has been demonstrated to occur in a small proportion of KD. Children with KD with macrophage activation syndrome tend to be older than those with KD without macrophage activation syndrome and have elevated markers of inflammation and end-organ dysfunction. Morbidity, including acute kidney injury, hepatic dysfunction, and acute respiratory distress syndrome is more frequent in those with KD with macrophage activation syndrome. Risk for mortality is also greater in these children.^5^

We appreciate Dr Wiwanitkit’s comment in which he rightfully states that while Covid-19-related KD should not be surprising that we should still acknowledge the difference of the KD in this setting. This is acknowledged and KD in the setting of Covid-19 has similar findings to previously described findings for KD with macrophage activation syndrome. Thus, it is likely that Covid-19 is proving to be a more potent and likely trigger of this rarer variety of KD.
